# The Impact of Transitions, a Mental Health Literacy Intervention With Embedded Life Skills for Postsecondary Students: Preliminary Findings From a Naturalistic Cohort Study

**DOI:** 10.1177/07067437211037131

**Published:** 2021-08-11

**Authors:** Yifeng Wei, Stan Kutcher, Erin Austen, Anne Comfort, Chris Gilham, Christiana MacDougall, Greg McKenna, Margaret McKinnon, Kara Thompson, Elizabeth Yeo, Michael Zhang, Andrew Baxter, Kara Matheson

**Affiliations:** 1 3158 University of Alberta, Edmonton, Alberta; 2 12361 Dalhousie University, Halifax, Nova Scotia; 3 1270 Saint Francis Xavier University, Antigonish, Nova Scotia; 4 7017 Mount Allison University, Sackville, New Brunswick; 5 113192 Holland College, Charlottetown, Prince Edward Island; 6 3690 Saint Mary’s University, Halifax, Nova Scotia; 7 3146 Alberta Health Services, Calgary, Alberta; 8 Nova Scotia Health Authority, Halifax, Nova Scotia

**Keywords:** mental health literacy, postsecondary institutions, stigma, stress, help seeking

## Abstract

**Objective:**

Mental illness is a common medical condition to onset during adolescence. Young people who leave for postsecondary life are at an especially challenging period of lifetime when many will leave home and familiar environments for prolonged periods of time. These new circumstances may put young people at risk of developing mental health problems or disorders or exacerbate existing mental disorders. Alternatively, some young people may misinterpret the normal negative emotional states occurring as a result of these new challenges as a mental disorder requiring professional intervention. We conducted a quasiexperimental cohort study to investigate the effectiveness of a mental health literacy intervention *Transitions* with blended life skills to address these challenges for first-year postsecondary students.

**Methods:**

Students (*n* = 2,397) from five Canadian postsecondary institutions were assigned to the intervention or the control group and were administered a survey at baseline, postintervention, and at 2-month follow-up (September 2017 to February 2018). We applied generalized linear mixed effects (PROC Mixed procedure) to test the between-group difference in the post—pre/follow-up—pre and to determine the predicted least-square mean values.

**Results:**

The findings showed that students who were exposed to the *Transitions* intervention significantly improved their mental health knowledge, decreased stigma against mental illness, improved help-seeking attitudes and behaviours, and decreased perceived stress when compared to students who had not been exposed to the intervention. However, we did not identify significant changes in general health. This may be due to the relatively short follow-up time (2 months) to determine participants’ general health status.

**Conclusions:**

*Transitions* delivered to first-year postsecondary students may be a beneficial intervention to help young people adjust to their new postsecondary life and improve their mental health.

## Abrégé

**Objetif:**

La maladie mentale est une affection médicale qui survient communément durant l’adolescence. Les jeunes gens qui partent vers une vie post-secondaire sont à une période spécialement difficile de leur vie car nombre d’entre eux quitteront la maison et des milieux familiers pour des périodes de temps prolongées. Ces nouvelles circonstances peuvent placer les jeunes à risque de développer des problèmes ou des troubles de santé mentale, ou exacerber les troubles mentaux existants. Par ailleurs, certaines jeunes gens peuvent interpréter à tort les états émotionnels négatifs normaux résultant de ces nouveaux défis comme étant un trouble mental nécessitant une intervention professionnelle. Nous avons mené une étude de cohorte quasi-expérimentale pour investiguer l’efficacité d’une intervention de littératie en santé mentale, *Transitions,* avec des compétences essentielles intégrées pour aborder ces problèmes de la première année des étudiants du post-secondaire.

**Méthodes:**

Les étudiants (n = 2 397) de 5 institutions post-secondaires canadiennes ont été affectés à l’intervention ou au groupe témoin où on leur a administré un sondage au départ, post-intervention et au suivi de 2 mois (septembre 2017-février 2018). Nous avons appliqué un modèle linéaire généralisé à effets mixtes (procédure PROC mixte) pour vérifier la différence entre les groupes dans le post – pré/suivi – pré changement et pour déterminer les valeurs moyennes prévues des moindres carrés.

**Résultats:**

Les résultats ont montré que les étudiants exposés à l’intervention *Transitions* ont amélioré significativement leurs connaissances en santé mentale; réduit les stigmates contre la maladie mentale; amélioré leurs attitudes et comportements face à la recherche d’aide; diminué le stress perçu quand ils étaient comparés avec les étudiants non exposés à l’intervention. Cependant, nous n’avons pas constaté de changements significatifs de la santé générale, ce qui peut être attribuable au temps relativement court du suivi (2 mois) pour déterminer l’état de santé général des participants.

**Conclusions:**

*Transitions* administrée aux étudiants de la première année post-secondaire peut être une intervention bénéfique pour aider les jeunes à s’adapter à leur nouvelle vie post-secondaire et à améliorer leur santé mentale.

## Introduction

Mental illness is the most common medical condition to onset during adolescence, with ∼50% of mental disorders onset by age 14 years and 75% by age 24 years.^1^ Furthermore, life-long health-promoting behaviours are often solidified by the end of the second decade of life.^2^ Postsecondary school students are therefore a cohort of interest and concern regarding mental health and mental illness.

Research indicated that 31% to 35% of postsecondary students were screened positive for at least one of the common lifetime/12-month disorders assessed.^3^ In Canada, the prevalence of mental disorders among youth aged 15 to 24 is estimated to be 10% to 15%.^4-6^ Mental disorders during adolescence have significant negative impacts, including impairment in daily functioning, intimate interpersonal relationship difficulties, poor learning outcomes and academic success, higher risk of alcohol and drug use, poorer vocational achievement, and reduced life expectancy.^7,8^ Suicide, commonly associated with mental disorders,^9,10^ is the second leading cause of death among youth.^11-13^ Despite the prevalence and life consequences of mental illness, help-seeking behaviours in this population are low, with estimates of 25.3% to 36.3% help-seeking rates for mental disorders and 29.5% to 36.1% for suicide thoughts and behaviours.^14^

Further, studies have identified that many young people in postsecondary institutions endorse a variety of negative emotions and coping challenges.^15-21^ These results have been often interpreted as a “mental health crisis” and an “epidemic of mental disorders” on campus with calls for more therapy and other mental health speciality services as a response,^22-27^ despite the fact that the prevalence of mental disorders among young people has been relatively stable over decades.^28-32^

These data reporting high rates of increases in perceived mental disorders in the face of stable prevalence suggest that some young people may not be able to distinguish normal existential stresses from a mental disorder due to a lack of mental health literacy.^33-35^ Similarly, this suggests that providing young people who are transitioning to postsecondary schooling with information that could enhance their mental health literacy while concurrently addressing the common and normal stress-inducing situations that they are likely to experience may be a useful mental health intervention. Mental health literacy entails four interrelated components: capacity to obtain and maintain good mental health; knowledge about mental health and mental disorders; stigma reduction; and help-seeking efficacy.^34,35^ A curriculum-integrated mental health literacy approach has been extensively applied and researched in secondary school settings to improve mental health literacy outcomes.^36-39^ However, the application of such curriculum-embedded approaches in postsecondary settings may not be feasible and alternative methods suitable for the particular contexts of postsecondary settings should be considered. This should be based on the reality of normal developmental challenges occurring during this time.

Many young people may head off to postsecondary institutions, leaving home for prolonged periods of time. These new circumstances create normal existential stresses, which may exacerbate existing mental disorders or demand coping strategies not yet developed. Concomitantly, young people may not realize that the difficulties they are experiencing could be due to a mental disorder rather than simply a reaction to life changes. Thus, they may not seek appropriate assistance with the result that early intervention and effective treatment do not occur expeditiously. Alternatively, young people may misinterpret the normal negative emotional states occurring as a result of these new challenges as a mental disorder requiring professional intervention. Therefore, providing information about mental health and mental illness in the context of other important issues that characterize this transition period may both enhance support to young people during this critical period and improve their ability to cope with these stresses, identify a mental disorder if it is on-setting and to seek help appropriately if needed.

This study investigated the impact of a mental health intervention Transitions that embeds mental health literacy into a comprehensive life skills resource for young people transitioning from secondary to postsecondary schooling. This most recent version of the Transitions updated the previous edition by including input from first-year university/college students, mental health experts, student-services staff, and faculty members. Building on previous research using earlier versions of Transitions that had demonstrated preliminary evidence on the improvements in mental health literacy,^40-42^ this current study applied a more robust research design with control groups in a large cohort that included both university and college students in five institutions of higher learning in Canada. This study hypothesized that mental health literacy embedded in a life skills resource for first-year students in postsecondary settings would promote student's understanding about mental health, improve their attitudes toward mental illness, reduce stigma against mental illness, improve their help-seeking efficacy and general health, and reduce perceived stress. This study was approved by the principal investigator's institution (# 2017-4174) and each of the participating institutions.

## Methods

### Participants and Recruitment

This study applied a quasiexperimental design with purposefully selected participants in each institution to participate in either the intervention group (participants who received Transitions) or the control group (participants who waited to receive Transitions). They were compared before Transitions was delivered, immediately after its delivery, and at 2-month follow-up. This design allowed the assignment of participants to the intervention or control group with similar demographic characteristics in this study.^43^ Given the significance level of α = 0.05, power 1 − β = 0.80, the number of predictors *n* = 4, and the medium effect size f2 = 0.15 for the multivariate analysis based on a similar study,^44^ we needed a total sample of *n* = 85 to achieve 80% power.

The study population was first-year undergraduate students enrolled in September 2017 from five institutions. We focused on Maritime provinces (Nova Scotia, New Brunswick, and Prince Edward Island) for study. We sent out invitation of the project participation to all universities and colleges across these three provinces and received confirmation of participation from five volunteering universities and colleges. We ended up recruiting over 2,000 students, representing ∼11% of the first-year university/college student population between 2017 and 2018 in these provinces, a quite large sample although it may not represent the whole student population in Canada. Each institution made its own decision on whether it would recruit students for both intervention or control groups or just for one study group depending on its contexts. Each institution designated specific staff to recruit participants.

At institutions 1 (*n* = 475) and 2 (*n* = 515), students were recruited by administrators within specific programs, intervention (*n* = 180 in institution 1; *n* = 265 in institution 2), and control (*n* = 295 in institution 1; *n* = 250 in institution 2) campuses were geographically distinct, thus decreasing the likelihood of cross-site contamination.

**Figure 1.**
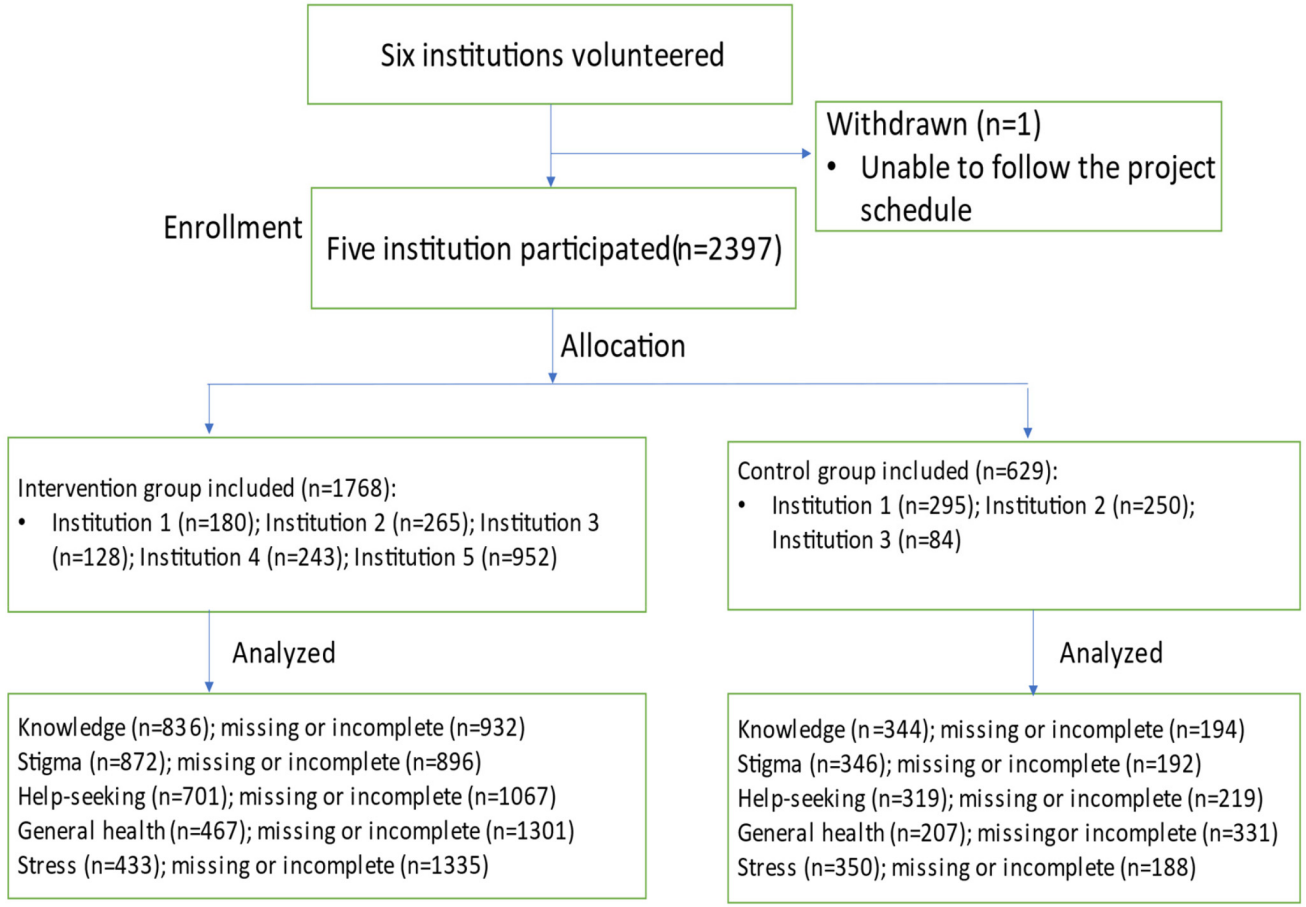
Recruitment process.

At institutions 3 to 5, faculty members who chose to participate in the study recruited students. At institution 3, 211 participants were drawn from a psychology course section (*n* = 84 control; *n* = 128 intervention). At institutions 4 (*n* = 243) and 5 (*n* = 952), students were drawn from various programs, all participating in the intervention only (see Figure 1).

### Procedure

Transitions (http://mentalhealthliteracy.org/product/transitions/) provides participants with information in five areas: information related to academic life, such as learning skills, class selection, preparing exams, and dealing with procrastination; knowledge about mental health and common mental disorders, stress management, treatment options, help-seeking resources, and sexual health; building multiple identities based on factors such as ethnicity, culture, gender, personality, values, spirituality and faith, and sexuality; building healthy relationships (e.g., friendship, romantic relationships, roommates, international students, and conflict resolution); and (mental) health help-seeking information and resources on campus. The delivery of Transitions was flexible depending on the context of the participating institution.

In all institutions, either a research assistant or a faculty member volunteering to facilitate the project administered an anonymous survey to students in both the intervention and control group at baseline. Then, the intervention group received a copy of Transitions and was given a 2-week period to read the Transitions resource. Reading took place after classes and in some cases, during classes. After this 2-week period, the research assistant or the faculty member administered the survey again, and at 2 months following the second survey in both the intervention and control groups. The study took place between September 2017 and February 2018.

Additionally, at institution 1, when students read Transitions, they were also provided with a short, in-classroom presentation by a research assistant that described Transitions and identified the different components in it. At institution 2, Transitions was provided to students as part of their course work, mostly assigned as readings for “homework” but the mental health portion was instructor-delivered in-class using a prepared presentation by a psychologist.

At institution 3, the intervention group participated in a 75-min in-class seminar, led by research assistants who developed a board game-*Thrive*-based on the Transitions content. At institutions 4 and 5, faculty members introduced Transitions in the classroom and students were asked to read Transitions after the class, and faculty members were available for support. We did not control how Transitions was implemented in each institution since it is impossible to implement such an intervention exactly the same manner in postsecondary institutions. The diversity of the delivery format evaluated in this study may inform other institutions to customize their implementation to maximize the intervention up-taking.

### Survey and Data Analysis

Five outcomes were observed: knowledge, stigma, help-seeking attitudes and behaviours, general health, and perceived stress (Appendix 1). Twenty knowledge questions investigated participants’ understanding about the content related to life skills and mental health. Participants chose one response (True, False, or Don't know) to each question. Each correct answer received a score of 1, and an incorrect answer, including “Don't know” received a score of 0. The total knowledge score was 20. The exploratory factor analysis demonstrated four factors (knowledge about mental health, mental disorders, and life challenges; stress and resilience; mental health treatment; and brain functions) addressing 42.45% of the variances with the current data. The internal consistency of the measure was α = 0.67 for the current data. We expected a relatively low internal consistency of the measure as it is a multidimensional scale covering various aspects of healthy life.

Twelve statements on stigma measured how participants perceived mental health and mental illness. Each statement had a score range of 1 to 7 on a Likert scale. Higher scores represented more positive attitudes, indicating lower stigma. Each participant received a total score between 7 and 84. The factor analysis identified one factor indicating the perceived stigma construct, with a total of 43.25% variances explained by the factor. The internal consistency reliability of the current data was α = 0.85.

For the attitudes toward help-seeking, there were five 7-point Likert scale statements with each response assigned a value between 1 and 7. Higher scores indicated more positive attitudes. Each participant received a total score of between 5 and 35. The factor analysis with the current data identified one factor indicating the help-seeking intentions construct, with a total of 69.29% variances explained by the factor. The internal consistency reliability of the current data was α = 0.81. Help-seeking behaviours were investigated with one question on whether participants in the past 2 months went to see a health professional for mental health problems or disorders.

For the general health outcome, there were 12 7-point Likert scale statements with each response assigned a value between 0 and 3. Higher scores indicated more severe health concerns. Each participant received a total score of between 0 and 36. General health measure has demonstrated strong reliability and validity in previous studies.^45^ The internal consistency reliability of the scale on the current data was α = 0.88.

For the perceived stress outcome, there were 10 7-point Likert scale statements with each response assigned a value between 0 and 4. Higher scores indicated more perceived stress. Each participant received a total score of between 0 and 40. The stress measure has demonstrated strong reliability and validity in previous studies.^46^ The internal consistency reliability of the scale on the current data was α = 0.79.

Descriptive statistics were reported as counts and percentages for categorical variables, means and standard deviation for normally distributed continuous variables, and medians and interquartile ranges for nonnormally distributed continuous variables. Baseline differences between the intervention group and control group were examined using chi-square test, Fisher's exact test, *t*-test, and/or Wilcoxon rank-sum test, as appropriate.

The dependent variable was the survey score for each of the five outcomes. Generalized linear mixed effects (PROC Mixed procedure) were used to test the between-group difference at each study time point and to determine the predicted least-square (LS) mean values. Study group (intervention or control) and time point of the survey were included in each model as fixed effects and institution as a random effect. Study group by time point interaction was included in each model as well as a condition with each additional demographic covariate including gender, previous mental health education, previous mental health treatment, and international student status. Variables and interactions found to be statistically significant at *P* < 0.1 in the univariate model analysis were included in the multivariate model.

The intraclass correlation coefficient (ICC) was applied to investigate variability in response across institutions. The log-linear analysis was applied to compare the number of students who opted to seek help at pretest and follow-up. All analyses were conducted using SAS/STAT software, with a significance level of α = 0.05.

## Results

Out of 2,397 students, 1,768 students were assigned to the intervention group and 629 students as controls (Figure 1). Of this, 1,183 students completed both pretest and posttest (49%); 939 students completed both pretest and 2-month follow-up surveys (40%). Of 2,397 students, 223 students reported help-seeking behaviours at both pretest and 2-month follow-up (9.3%). The pre–post analysis measured: knowledge, stigma, and help-seeking attitudes. The pretest and 2-month follow-up analysis evaluated: help-seeking behaviours, general health, and perceived stress as it took time to measure changes in these outcomes. Generalized linear mixed models were used to model the data with data points entered into the models separately at pre and post time points. This enables data points to be included in the model whether or not the student reported answers at each time point thus reducing the amount of information lost.

Table 1 presents participants’ demographics. A chi-square test revealed significant differences between group by gender, with significantly more female participants (56.7%) than expected in the Transitions group (χ^2^(1) = 5.14, *P* = 0.02); by student nationality, with significantly more Canadian participants (93.2%) than expected in the control group (χ^2^(1) = 63.83, *P* = 0.000); and by previous treatment of oneself or their family members, with significantly more participants (49.3%) than expected who had previous treatment in the control group (χ^2^(1) = 6.41, *P* = 0.01); however, not by previous mental health education (χ^2^(1) = 2.03, *P* = 0.15). Demographic differences were factored in the PROC mixed model and controlled in the data analysis.

**Table 1.** Demographics.

Variables	Overall		Transitions (*n* = 1,768)		Control (*n* = 629)		*X* ^2^	*p*
	*n* = 2,397	(%)	Actual *n* (%)	Expected *n* (%)	Actual *n* (%)	Expected *n* (%)		
Gender								
Male	1,022	44.7	735 (43.3)	758 (44.7)	287 (48.7)	263 (44.7)	5.14	0.02*
Female	1,263	55.3	961 (56.7)	937 (55.3)	302 (51.3)	325 (55.3)		
Previous mental health education								
Yes	579	25.1	417 (24.4)	430 (25.1)	162 (27.3)	149 (25.1)	2.03	0.15
No	1,725	74.9	1294 (75.6)	1,281 (74.9)	431 (72.7)	444 (74.9)		
Previous mental health treatment for oneself or family members								
Yes	966	44.7	689 (43.1)	714 (44.8)	277 (49.3)	251 (44.8)	6.41	0.01*
No	1,194	55.3	909 (56.9)	883 (55.2)	285 (50.7)	310 (55.2)		
Nationality								
Canadian	1,912	82.6	1350 (78.9)	1414 (82.6)	562 (93.2)	498 (82.6)	63.83	0.000*
Not Canadian	403	17.4	362 (21.1)	298 (17.4)	41 (6.8)	105 (17.4)		

*P < 0.05.

An ICC analysis indicated nonsignificant institution differences regarding knowledge (ICC = 0.078), stigma (ICC = 0.006), help-seeking attitudes (ICC = 0.013), general health (ICC = 0.037), or stress (ICC = 0.156).

### Knowledge

The univariate analysis of gender (*p* = 0.85) was not statistically significant and therefore gender was excluded in the multivariate model.

A multivariate analysis identified a statistically significant interaction between the groups and the knowledge score (*P* < 0.001). The LS mean estimate was calculated at three different mean population pretest scores: 5, 10, and 15. The LS mean difference estimate was statistically significant when comparing intervention group post to pre knowledge total, 1.85 (95% CI, 1.68 to 2.02). The intervention group estimate was 1.878 (95% CI, 1.53 to 2.22) higher post follow-up compared to the control group. There was no statistically significant difference between the intervention and control group at baseline, (experimental–control) −0.11 (95% CI, −0.43 to 0.21).

There was a statistically significant difference between those with previous mental health treatment and those without, 1.05 (95% CI, 0.83 to 1.26, *P* < 0.001). Those with previous mental health education had a higher LS mean estimate of knowledge score compared to those without previous mental health education, 0.49 (95% CI, 0.27 to 0.73, *P* < 0.001), and Canadian students had a higher LS mean estimate of the knowledge score, 1.72 (95% CI, 1.43 to 2.0,1 *P* < 0.001), compared to those who were international visa students.

### Stigma

The univariate analysis demonstrated that demographics significantly predicted the pre–post change of stigma scores. A multivariate analysis demonstrated that the LS mean estimate in stigma was 1.22 (95% CI, 0.04 to 2.40, *P* = 0.042) greater for the intervention group compared to the control. There was no statistically significant difference between intervention and control at baseline, 0.114. This demonstrates greater improvements in stigma reduction in the intervention group.

It also showed no statistically significant interaction between gender and study groups, *P* = 0.136. However, there was a statistically significant difference in stigma LS difference estimate for women compared to men, 3.82 (95% CI, 2.64 to 4.99).

Pretest stigma, previous mental health treatment, previous mental health education, and international student status did not interact with the study groups, and therefore did not impact the pre–post change in stigma scores between the intervention and control groups (*P* > 0.05).

### Help-Seeking Attitudes and Behaviours

The univariate modeling for help-seeking attitudes and international student status was not statistically significant (*P* = 0.75). A multivariate analysis demonstrated that the intervention group had an LS mean difference estimate of post–pre score 1.287 (95% CI, 0.94 to 1.63, *P* < 0.001) compared to 0.77 (95% CI, 0.24 to 1.30, *P* = 0.004) in the control group. The LS mean estimate for those with previous mental health treatment was 1.12 (95% CI, 0.68 to 1.57, *P* < 0.001) greater than for those without previous treatment (*P* = 0.011). Females had a greater LS mean estimate of 0.682 (95% 0.25, 1.11, *P* = 0.002) compared to males and those with previous mental health education has a greater LS estimate of 0.59 (95% CI, 0.12 to 1.06, *P* = 0.013).

We applied the three-way log-linear analysis (condition × pretest × follow-up) to observe the number of students who opted for help (either already spoke to a health provider or waited to see a health provider) against the number of students who did not at pretest and 2-month follow-up. Although the three-way interaction was not significant (χ^2^(1) = 1.45, *P* > 0.05), the two-way chi-square test indicated at 2-month follow-up there was a significant association between the group type (intervention or condition group) and help-seeking behaviours (χ^2^(1) = 20.65, *P* < 0.001). Based on the odds ratio, the odds of students in the intervention group seeking help were 2.75 times greater than students in the control group at the 2-month follow-up.

### General Health

A univariate analysis showed no statistically significant association between post and prechange in general health with the study groups (*P* = 0.762) implying that the intervention did not influence the general health outcome among participants.

### Perceived Stress

A univariate analysis showed no statistically significant association between study group and gender (*P* = 0.1429), previous mental health treatment (*P* = 0.5429), international student visa status (*P* = 0.77), and previous mental health education (*P* = 0.11).

The impact of the intervention varied depending on the time point. In the intervention group, the LS mean difference estimate (pre–post) was 3.451 (95% CI, 2.92 to 3.99, *P* < 0.001), compared to −0.64 (95% CI, −1.28 to −0.002, *P* = 0.049) in the control group.

## Discussion

This is the first large-scale controlled study of an intervention blending life skills information with mental health literacy for postsecondary youth that addressed campus mental health and life. The findings showed that students in the intervention significantly improved mental health knowledge, decreased stigma against mental illness, increased positive attitudes toward help-seeking, improved help-seeking behaviours, and decreased perceived stress compared to the control group. However, we did not identify significant changes in the general health outcome. This may be due to the relatively short follow-up time (2 months) to determine participants’ general health status, implying the extension of follow-up time for this measure in the future.

These findings are consistent with the previous research on earlier versions of the Transitions, reporting it to be a helpful resource with demonstrated benefits for student postsecondary life.^40-42,47^ The findings described herein not only replicated those findings in short term and further at 2-month follow-up using a more robust research design on more validated outcomes across different institutions. The finding that students who had lower mental health knowledge scores at baseline improved most indicates that it may be especially beneficial to students with a poor understanding about mental health. Students also demonstrated greater help-seeking behaviours for mental health problems, which supports the potential clinical relevance of this finding because students may be more knowledgeable about their mental health status and about where to seek appropriate services, resulting in potential early identification and treatment of mental disorders. Furthermore, this study first demonstrated significantly less stress in the intervention group compared to the control group. This is consistent with approaches suggested by other researchers^48,49^ and addressed the challenges that young people endorse a variety of negative emotions and coping challenges during the transition period to postsecondary life.^15-21^ These results suggest that the application of the Transitions may be of benefit in addressing mental health in postsecondary students.

Although each institution applied Transitions differently, institutions performed similarly on outcomes. This indicates that Transitions may be delivered using flexible formats and it allows for campuses to tailor its implementation so that it can be integrated into different campus culture and context. However, some delivery methods may provide enhanced outcomes. For example, including mental health providers in delivery of the mental health sections may enhance outcomes.

We found that gender, previous mental health education, or previous treatment experience did not influence how participants performed on all outcomes except for gender difference on the stigma and previous mental health treatment on the knowledge outcome. This may imply that exposure to Transitions will produce a similar impact on students regardless of these demographics. However, students with aboriginal, African, and immigrant/international background were not well represented in this sample, warranting further studies with participants of diverse backgrounds.

The study did not apply a randomized controlled design and we chose the control group participants purposefully, which may have biased the study results. The control group included students mainly from two colleges and one university campus and may have not included adequate samples from university settings, leading to potential bias of the results. Further, the relatively high attrition rate at a 2-month follow-up may have biased the longer-term results and limited its generalizability.

## Conclusions

While mental health literacy could be an important component of a campus-wide approach to mental health^50^—this has, to date not been widely applied due to a dearth of evidence-based interventions, even if policies exist.^51-55^ Thus, Transitions provides a tool that could be used as an evidence-based solution to campus mental health concerns raised in public.^56,57^

## Supplemental Material

sj-sav-1-cpa-10.1177_07067437211037131 - Supplemental material for The Impact of Transitions, a Mental Health Literacy Intervention With Embedded Life Skills for Postsecondary Students: Preliminary Findings From a Naturalistic Cohort Study Click here for additional data file. Supplemental material, sj-sav-1-cpa-10.1177_07067437211037131 for The Impact of Transitions, a Mental Health Literacy Intervention With Embedded Life Skills for Postsecondary Students: Preliminary Findings From a Naturalistic Cohort Study by Yifeng Wei, Stan Kutcher, Erin Austen, Anne Comfort, Chris Gilham, Christiana MacDougall, Greg McKenna, Margaret McKinnon, Kara Thompson, Elizabeth Yeo, Michael Zhang, Andrew Baxter and Kara Matheson in The Canadian Journal of Psychiatry

## References

[1] Kessler RC Berglund P Demler O , et al. Lifetime prevalence and age-of-onset distributions of DSM-IV disorders in the national comorbidity survey replication. Arch Gen Psychiatry. 2005;62(6):593-602. 15939837 10.1001/archpsyc.62.6.593

[2] World Health Organization. Global accelerated action for the health of adolescents (AA-HA!): guidance to support country implementation. 2018. Available from: https://apps.who.int/iris/bitstream/handle/10665/255415/9789241512343-eng.pdf?sequence=1

[3] Auerbach RP Mortier P Bruffaerts R , et al. WHO world mental health surveys international college student project: prevalence and distribution of mental disorders. J Abnorm Psychol. 2018;127(7):623-638 30211576 10.1037/abn0000362 PMC6193834

[4] Standing Senate Committee on Social Affairs, Science and Technology. Report 1 – Mental health, mental illness and addiction: Overview of policies and programs in Canada. November 2004; Chapter 5, Section 5.1.2, 86.

[5] Government of Canada. The human face of mental health and mental illness in Canada. Ottawa: Minister of Public Works and Government Services Canada, 2006.

[6] Waddell C Shephard C Schwartz C , et al. Child and youth mental disorders: prevalence and evidence-based interventions. A research report for the British Columbia Ministry of Children and Family Development. 2014.

[7] Bhatia S . Childhood and adolescent depression. Am Fam Physician. 2007;75(1):73. 17225707

[8] Kessler RC Foster CL Saunders WB , et al. Social consequences of psychiatric disorders: educational attainment. Am J Psychiatry. 1995;152(7):1026-1032. 7793438 10.1176/ajp.152.7.1026

[9] Mann JJ . A current perspective of suicide and attempted suicide. Ann Intern Med. 2002;136(4):302-311. 11848728 10.7326/0003-4819-136-4-200202190-00010

[10] Zalsman G Hawton K Wasserman D , et al. Suicide prevention strategies revisited: 10-year systematic review. Lancet Psychiatry. 2016;3(7):646-659. 10.1016/S2215-0366(16)30030-X 27289303

[11] Canadian Psychiatric Association. Mental Illness Awareness Week fact sheet. Available from: http://www.ontario.cmha.ca/fact_sheets.asp?cID=3965 (Statistics Canada (n.d.) Table 102-0551 Deaths, by selected grouped causes, age group and sex, Canada, provinces and territories, annual (table). CANSIM (Database). 2002. Version updated November 30, 2006.

[12] Government of Canada. Suicide in Canada: Key statistics. 2019. Available from: https://www.canada.ca/content/dam/phac-aspc/documents/services/publications/healthy-living/suicide-canada-key-statistics-infographic/pub-eng.pdf

[13] World Health Organization. WHO Global Health Estimates. 2016. Available from: http://www.who.int/healthinfo/global_burden_disease/esitmates

[14] Bruffaerts R Mortier P Auerbach RP , et al. Lifetime and 12-month treatment for mental disorders and suicidal thoughts and behaviors among first year college students. Int J Methods Psychiatr Res. 2019; 28(2):e1764. [Epub ahead of print 2019 Jan 20]. 30663193 10.1002/mpr.1764 PMC6877191

[15] Blanco C Okuda M Wright C , et al. Mental health of college students and their non-college-attending peers: results from the national epidemiologic study on alcohol and related conditions. Arch Gen Psychiatry. 2008;65(12):1429-1437. 19047530 10.1001/archpsyc.65.12.1429 PMC2734947

[16] Gruttadaro D Crudo D , for National Alliance on Mental Illness. College students speak: A survey report on mental health. 2012. Available from: https://www.nami.org/getattachment/About-NAMI/Publications-Reports/Survey-Reports/College-Students-Speak_A-Survey-Report-on-Mental-Health-NAMI-2012.pdf

[17] Eisenberg D Lipson SC . The Healthy Minds Study: 2016–2017 data report. 2018. Available from: https://wellbeing.humboldt.edu/sites/default/files/health/shws_data_2017_healthyminds_report_humboldt.pdf

[18] NatCen for Mental Health Foundation. Surviving or thriving? The State of the UK’s mental health. 2017. Available from: https://www.mentalhealth.org.uk/sites/default/files/surviving-or-thriving-state-uk-mental-health.pdf

[19] Patalay P Fitzsimons E . Mental ill-health among children of the new century: trends across childhood with a focus on age 14. 2017. Centre for Longitudinal Studies: London. Available from: https://www.ncb.org.uk/sites/default/files/uploads/documents/Research_reports/UCL%20-%20NCB%20-%20Mental_Ill-Health%20FINAL.pdf

[20] National Institute of Mental Health. Mental health information statistics. Bethesda, MD: National Institute of Mental Health; 2019. Available from: https://www.nimh.nih.gov/health/statistics/mental-illness.shtml

[21] Lipson SK Lattie EG Eisenberg D . Increased rates of mental health service utilization by U.S. college students: 10-Year population-level trends (2007–2017). 2018. 10.1176/appi.ps.201800332 PMC6408297 30394183

[22] Lunau K . The mental health crisis on campus. 2012. Available from: https://www.macleans.ca/education/uniandcollege/the-mental-health-crisis-on-campus/

[23] Henriques G . The college student mental health crisis. 2014. Available from: https://www.psychologytoday.com/ca/blog/theory-knowledge/201402/the-college-student-mental-health-crisis

[24] Eiser A . The crisis on campus: APA is working with congress to address serious mental health problems on college campuses. 2011. Available from: https://www.apa.org/monitor/2011/09/crisis-campus

[25] Shackle S . The way universities are run is making us ill: inside the student mental health crisis. 2019. Available from: https://www.theguardian.com/society/2019/sep/27/anxiety-mental-breakdowns-depression-uk-students

[26] National Children’s Bureau. One in four girls is depressed at age 14, new study reveals. 2017. Available from: https://www.ncb.org.uk/news-opinion/news-highlights/one-four-girls-depressed-age-14-new-study-reveals

[27] Mental Health Foundation. Stressed nation: 74 per cent of UK ‘overwhelmed or unable to cope’ at some point in the past year. 2018. Available from: https://www.mentalhealth.org.uk/news/stressed-nation-74-uk-overwhelmed-or-unable-cope-some-point-past-year

[28] Merikangas KR He JP Burstein M , et al. Lifetime prevalence of mental disorders in US adolescents: results from the national comorbidity study-adolescent supplement (NCS-A). J Am Acad Child Adolesc Psychiatry. 2010;49(10):980-989. 20855043 10.1016/j.jaac.2010.05.017 PMC2946114

[29] NHS Digital, Governmental Statistical Service. Mental health of children and young people in england, 2017: summary of key findings. Available from: https://files.digital.nhs.uk/F6/A5706C/MHCYP%202017%20Summary.pdf

[30] Wiens K Bhattarai A Dores A , et al. Mental health among Canadian postsecondary students: a mental health crisis? Can J Psychiatry [Internet]. 2019. 10.1177/0706743719874178 PMC6966257 31939333

[31] Patten SB Williams JVA Lavorato DH , et al. The prevalence of major depression is not changing. Can J Psychiatry [Internet]. 2015;60(1):31-34. Available from: https://www.ncbi.nlm.nih.gov/pmc/articles/PMC4314054/pdf/cjp-2015-vol60-january-31-34.pdf 10.1177/070674371506000107 PMC4314054 25886547

[32] Comeau J Georgiades K Duncan L , et al. Changes in the prevalence of child and youth mental disorders and perceived need for professional help between 1983 and 2014: evidence from the ontario child health study. Can J Psychiatry. 2019;64(4):256-264. 10.1177/0706743719830035 30978139 PMC6463358

[33] American College Health Association. American College health association-national college health assessment II: canadian reference group data report spring 2019. Silver Spring, MD: American College Health Association; 2019.

[34] Kutcher S Wei Y Costa S , et al. Enhancing mental health literacy in young people. Eur Child Adolesc Psychiatry. 2016;25(6):567–569. 27236662 10.1007/s00787-016-0867-9

[35] Kutcher S Wei Y Coniglio C . Mental health literacy: past, present, and future. Can J Psychiatry. 2016;61(3):154-158. 27254090 10.1177/0706743715616609 PMC4813415

[36] Morgan AJ Ross A Reavley NJ . Systematic review and meta-analysis of mental health first aid training: effects on knowledge, stigma, and helping behavior. PLoS One. 2018;13(5) e0197102. 29851974 10.1371/journal.pone.0197102 PMC5979014

[37] Milin R Kutcher S Lewis S , et al. Impact of a mental health curriculum on knowledge and stigma among high school students: a randomized controlled trial. J Am Acad Child Adolesc Psychiatry. 2016;55(5):383-391. 27126852 10.1016/j.jaac.2016.02.018

[38] Kutcher S Wei Y Morgan C . Successful application of a Canadian mental health curriculum resource by usual classroom teachers in significantly and sustainably improving student mental health literacy. Can J Psychiatry. 2015;60(12):580-586. 26720827 10.1177/070674371506001209 PMC4679167

[39] McLuckie A Kutcher S Wei Y , et al. Sustained improvements in students’ mental health literacy with use of a mental health curriculum in Canadian schools. BMC Psychiatry. 2014;14(1):379. 25551789 10.1186/s12888-014-0379-4 PMC4300054

[40] Potvin-Boucher J Szumilas M Sheikh T , et al. Transitions: a mental health literacy program for postsecondary students. J Coll Stud Dev. 2010;51(6):723-727.

[41] Kutcher S Wei Y Morgan C . Mental health literacy in post-secondary students. Health Educ J. 2015;75(6):689-697.

[42] Hunt S Wei Y Kutcher S . Addressing mental health literacy in a UK university campus population: positive replication of a Canadian intervention. Health Educ J. 2019;78(5):537-544.

[43] Oleckno WA . Essential epidemiology: principles and applications. Long Grove, Illinois: Waveland Press. 2002.

[44] Carr W Wei Y Kutcher S , et al. Mental health literacy in pre-service teachers. Can J School Psychol. 2017;33(4):314-326.

[45] Hankins M . The reliability of the twelve-item general health questionnaire (GHQ-12) under realistic assumptions. BMC Public Health. 2008;8:355. 10.1186/1471-2458-8-355 18854015 PMC2572064

[46] Roberti JW Harrington LN Storch EA . Further psychometric support for the 10-item version of the perceived stress scale. J Coll Counseling. 2006;9(2):135-147.

[47] Gilham C Austen E Wei Y , et al. Improving mental health literacy in post-secondary students: field testing the feasibility and potential outcomes of a peer led approach. Can J Commun Ment Health. 2018;37(1):1-12.

[48] McGonigal K . The upside of stress: why stress is good for you, and how to get good at it. Avery, 2016.

[49] Harvard University Centre of the Developing Child. Toxic stress. Available from: https://developingchild.harvard.edu/science/key-concepts/toxic-stress/

[50] Eells GT Marchell TC Corson-Rikert J , et al. A public health approach to campus mental health promotion and suicide prevention. Harvard Health Policy Rev. 2012;13:3-6. Available from: http://www.hcs.harvard.edu/∼hhpr/wp-content/uploads/2012/04/features-1.pdf

[51] De Somma E Jaworska N Heck E , et al. Campus mental health policies across Canadian regions: need for a national comprehensive strategy. Can Psychol/Psychol Can. 2017;58(2):161-167. 10.1037/cap0000089

[52] Came H Tudor K . The whole and inclusive university: a critical review of health promoting universities from New Zealand. Health Promot Int. 2020;35:102-110. 30590477 10.1093/heapro/day091

[53] Dooris M Farrier A Holt M , et al. Whole system approaches to health in higher education: an evaluation of the UK healthy universities network. Health Educ. 2019;119(4):246-258. Available from: https://www.emerald.com/insight/content/doi/10.1108/HE-02-2019-0010/full/html?af=R

[54] Suárez-Reyes M Van den Broucke S . Implementing the health promoting university approach in culturally different contexts: a systematic review. Glob Health Promot. 2016;23(Suppl. 1):46-56. 27199017 10.1177/1757975915623933

[55] Cawood J Dooris M Powell S . Healthy universities: shaping the future. Perspect Public Health. 2016;130(6):259-260. 10.1177/1757913910384055 21213559

[56] Canadian Association of College & University Student Services and Canadian Mental Health Association. Post-Secondary student mental health: guide to a systemic approach. Vancouver, BC: Canadian Association of College & University Student Services and Canadian Mental Health Association, 2013.

[57] Best Practices in Canadian Higher Education. An environmental scan of Canadian campus mental health strategies. Toronto, ON: Best Practices in Canadian Higher Education, 2019.

